# Solitary functioning kidney in children: clinical implications

**DOI:** 10.1590/1678-4685-JBN-3942

**Published:** 2018-06-18

**Authors:** Veerbhadra Radhakrishna, Krishna Kumar Govindarajan, Kumaravel Sambandan, Bibekanand Jindal, BikashKumar Naredi

**Affiliations:** 1 Jawaharlal Institute of Postgraduate Medical Education & Research, Department of Pediatric Surgery, Pondicherry, 605006, India.

**Keywords:** Kidney Diseases, Repertory: Kidneys Section, Kidney, Congenital Abnormalities, Renal Insufficiency, Nefropatias, Repertório: Seção Rins, Rim, Anormalidades Congênitas, Insuficiência Renal

## Abstract

**Introduction:**

Children with solitary functioning kidney (SFK) are prone to develop long term problems, which are not well represented in the literature. The extent to which the presence of associated congenital anomalies of kidney and urinary tract (CAKUT) further de-stabilize renal function is to be addressed.

**Objective:**

This study was conducted to evaluate the etiology, presentation, presence of CAKUT, and renal damage in children with SFK.

**Methods:**

All children with SFK who presented to the department of pediatric surgery from March 2014 to May 2016 were included in the study. Children with malignancy were excluded from the study.

**Results:**

Of the 20 patients with SFK, 14 (70%) had primary SFK (8 with agenesis and 6 with multicystic dysplastic kidney), 6 (30%) belonged to secondary SFK group, among them 3 had pelviureteric junction obstruction, 2 had posterior urethral valves and 1 had vesicoureteric reflux. Eight (40%) had associated CAKUT, 4 (20%) were asymptomatic while 8 (40%) had UTI and 6 (30%) had hypertension. Ten (50%) patients had reduced glomerular filtration rate (GFR) suggesting compromised renal function.

**Conclusion:**

Children with SFK have high morbidity especially when associated with ipsilateral CAKUT. Long-term periodical follow up is essential in these patients to improve clinical outcome.

## RESUMO

**Introdução:**

Crianças com rim solitário funcional (RSF) tendem a desenvolver problemas de longo prazo, que não são bem representados na literatura. Devemos abordar o quanto a presença de malformações congênitas do rim e do trato urinário (CAKUT) desestabiliza ainda mais a função renal.

**Objetivo:**

Este estudo foi realizado para avaliar a etiologia, apresentação, presença de CAKUT e dano renal em crianças com RSF.

**Métodos:**

Todas as crianças com RSF que se apresentaram no departamento de cirurgia pediátrica de março de 2014 a maio de 2016 foram incluídas no estudo. Crianças com doença maligna foram excluídas do estudo.

**Resultados:**

dos 20 pacientes com RSF, 14 (70%) tinham RSF primário (8 com agenesia e 6 com rim displásico multicístico), 6 (30%) pertenciam ao grupo RSF secundário, dentre eles 3 tinham obstrução da junção pelveuretérica, 2 tinham valvas uretrais posteriores e 1 refluxo vesico-uretral. Oito (40%) tinham ACRTU associado, 4 (20%) estavam assintomáticos, enquanto 8 (40%) tinham ITU e 6 (30%) tinham hipertensão. Dez (50%) pacientes apresentaram redução da taxa de filtração glomerular (TFG), sugerindo comprometimento da função renal.

**Conclusão:**

Crianças com RSF apresentam alta morbidade, especialmente quando associadas à CAKUT ipsilateral. O acompanhamento periódico a longo prazo é essencial nesses pacientes para melhorar o resultado clínico.

## INTRODUCTION

It was believed until a few years ago that one could live just as well with one kidney as with two. This might hold well for adults but not for children. Recent studies have revealed that children with a solitary functioning kidney (SFK) are prone to develop proteinuria, hypertension, glomerulosclerosis and chronic kidney disease (CKD). SFK can be primary (pSFK) if it is due to contralateral renal agenesis, aplasia or multicystic kidney disease, or secondary (sSFK) if it is due to one of the congenital anomalies of kidneys or from urinary tract infection destroying the contralateral kidney^1^. Children with pSFK adapt better than sSFK due to the early compensatory changes and having more nephrons. In spite of this, 50% of children with pSFK need dialysis by 30 years of age^2^. Hence, a study was undertaken to evaluate the etiology, associated anomalies including CAKUT, and status of renal function in children with SFK.

## OBJECTIVES

1. To analyze the etiology, clinical status, and renal functional status in children with SFK.
2. To compare primary SFK (pSFK) with secondary SFK (sSFK).
3. To determine the effect of ipsilateral congenital anomalies of kidney and urinary tract (CAKUT) in SFK.

## DESIGN AND METHODS

A prospective study was conducted in the Department of Pediatric Surgery, JIPMER, India, for 26 months from March 2014 to May 2016. All children with SFK presented during the study period were included. Children with malignancy were excluded.

The nature of the study was explained to the children and their parents, and a written informed consent was obtained from parents. Children older than 7 years of age gave assent to be included in the study.

A medical history was taken, clinical examination was carried out, and findings were noted in a form. Length/height and weight were matched with the Indian Association of Pediatrics reference charts.^3^ Wasting was defined as the weight ≤ 3rd centile and Stunting as the length/height ≤ 3rd centile. Blood pressure (BP) was measured using non-invasive blood pressure (NIBP) monitor. A child was considered hypertensive if at least three readings of systolic/diastolic/mean arterial pressure were more than or equal to 95th centile for age, sex, and height. The upper right arm was used to standardize and compare with standard tables^4^ and avoid error if the child had coarctation of aorta. Neonates, infants, and young children underwent sleeping BP while the older children were seated with their back supported, feet on the floor, and right arm supported with cubital fossa at the level of heart. BP cuff was selected with inflatable bladder width at least 40% of the circumference of the arm at a midway point between the olecranon and the acromion, with the cuff covering about 80-100% of the circumference of the arm.

Blood samples were collected from a peripheral venipuncture and urine was collected by mid-stream clean catch or from catheter if the patient was catheterized for any other purpose. Microalbuminuria was estimated using ELISA kit. Urine albumin level >30mg/L was considered significant. Serum and urine electrolytes were measured by ion selective electrode (ISE) method. Estimated glomerular filtration rate (eGFR) was calculated by Modified Schwartz equation.^5^

GFR (mL/min/1.73 m^2^) = (0.41 × Height in cm) / Creatinine in mg/dL

The eGFR was matched against normal range for the age defined by Holliday et al.^6^ (Table 1). Chronic kidney disease (CKD) was defined as per KDIGO 2012 Clinical Practice Guidelines.^7^ For children older than 2 years, CKD was defined as GFR less than 60 mL/min/1.73m^2^ or the presence of at least one of the markers of kidney damage (albuminuria, urine sediment abnormalities, electrolyte abnormalities, and structural abnormalities defined by imaging) persisting for more than 3 months. For children younger than 2 years, GFR less than the age normative value as defined by Holliday et al^6^ was used for defining CKD.

**Table 1:** Normal GFR values.

AGE	Mean GFR (mL/min/m^2^)	Range (mL/min/m^2^)
Neonate <34 weeks of gestation		
2-8 days	11	11-15
4-28 days	20	15-28
30-90 days	50	40-65
Neonates >34 weeks of gestation		
2-8 days	39	17-60
4-28 days	47	26-68
30-90 days	58	30-86
1-6 months	77	39-114
6-12 months	103	49-157
12-19 months	127	62-191
>2 years	127	89-165

Statistical methods:

- The various demographical parameters, clinical features, and abnormal laboratory findings are reported in percentages.
- The differences between various groups were analyzed using chi-square test. Two groups were considered significantly different when the p value was <0.05 (CI- 95%).

## RESULTS

A total of 45 patients with SFK was studied. The mean age of the study group was 4.04 ± 3.69 years. The youngest patient was 5 days old while a 14-year-old child was the oldest. Thirty-four (76%) were males and 11 (24%) were females, 31 (69%) belonged to pSFK while 14 (31%) belonged to sSFK. The most common cause of pSFK was unilateral renal agenesis/aplasia (URA) (20 children, 65%) while pelvi-ureteric junction obstruction (PUJO) was the most common cause of sSFK (6 children; 43%, Table 2). Twenty-six (58%) had left SFK and 19 (42%) had right SFK; 13 (29%) patients had ipsilateral CAKUT. Among those with ipsilateral CAKUT, vesico-ureteric reflux (VUR) was seen in 10 (77%) children, PUJO in 2 (15%) children, and obstructive megaureter in one child. Seventeen (38%) had associated non-urinary anomalies, 10 (48%) had anorectal malformation, 3 (7%) had undescended testis, 2 (4%) had cardiac anomalies and one each had congenital glaucoma, sacral agenesis, preauricular tags, inguinal hernia, and umbilical adenoma.

**Table 2:** Causes of solitary functioning kidney.

pSFK	Number (%)	sSFK	Number (%)
Unilateral renal agenesis/aplasia (URA)	20 (64.5%)	Pelvi-ureteric junction obstruction (PUJO)	6 (42.9%)
Multicystic dysplastic kidney (MCDK)	11 (35.5%)	Posterior urethral valves (PUV)	5 (35.7%)
		Vesico-ureteric reflux (VUR)	3 (21.4%)

Fourteen (31%) patients were asymptomatic while the same number of patients developed urinary tract infection. Eight children (18%) had abdominal pain, 6 (13%) had urosepsis, 5 (11%) had abdominal mass and dribbling of urine and 2 (4%) had hematuria. Eleven (24%) children were found to have hypertension, 15 (33%) were wasted, 14 (31%) stunted while 12 (27%) were both wasted and stunted. The patients with albuminuria, reduced eGFR, and CKD were 27 (60%), 31 (69%) and 18 (40%) respectively. Forty-one (91%) had either hypertension, albuminuria or reduced eGFR while 6 (13%) had all three parameters.

Patients with sSFK were found to have significantly higher rate of albuminuria (12/14 vs 15/31; p=0.02) compared to pSFK but no statistical difference was found in terms of hypertension (3/14 vs 8/31; p=0.75), reduced eGFR (10/14 vs 21/31; p=0.8) and CKD (6/14 vs 12/31; p=0.4).

Patients with SFK and ipsilateral CAKUT were found to have higher rates of hypertension (6/13 vs 5/32; p=0.03), reduced eGFR (12/13 vs 19/32; p=0.03), CKD (10/13 vs 8/32; p=0.06) and albuminuria (12/13 vs 15/32; p=0.005) compared to SFK without ipsilateral CAKUT.

## DISCUSSION

In experimental studies, Brenner et al.^8^ emphasized that reduction in renal mass leads to glomerular hyperfiltration and hypertrophy, and systemic hypertension. They suggested congenital nephron endowment is a major factor in the pathogenesis of CKD. This raised apprehensions about the long-term outcome of SFK. Ibrahim et al.^9^ studied 3698 live renal donors between 1963 and 2007 for long-term outcome of SFK and found that prevalence of end-stage renal disease (ESRD) was lower than that of the general population. The favorable result in that study was probably due to the rigid criteria to select renal transplant donors and because the long-term outcome in adults is different from that in children. Children with SFK are different from adults with SFK regarding genetics, nephron endowment, influence of intrauterine environment, and associated CAKUT. These facts are supported by Keller et al^10^ and Hughson et al^11^.

Westland et al.^1^ and Sanna-Cherchi et al.^2^ showed that males are more commonly affected by SFK than females and our study supported this finding.

In our sample, 69% had primary SFK. Unilateral renal agenesis/aplasia is the most common cause of SFK, which constituted 65% of the pSFK group. Unilateral renal agenesis (URA) occurs due to the failure of metanephric blastema induction by the ureteral bud. It is seen in 1 in 1100 autopsies with a slight male and left-sided preponderance.^12^

The other important cause of pSFK is multicystic dysplastic kidney (MCDK), which is the most common renal cystic disease and is mostly unilateral. It is seen in 1 in 2500 neonates and is associated with contralateral vesico-ureteric reflux (VUR) in 4-31% of the cases, which explains the association of renal failure and hypertension with MCDK. In addition, literature indicates that there is a predisposition of MCDK to Wilms' tumor.^13^

The most common cause of secondary SFK in our study was PUJO, which occurs due to failure or insufficient canalization of pelviureteric junction (PUJ).^14^ It is also thought to occur due to defective smooth muscle cells at PUJ with improper neural innervation leading to functional obstruction. The persistent obstruction at the PUJ leads to compression and ischemic necrosis of renal papillae, injury to the loop of Henle, tubular dilatation, glomerulosclerosis, inflammatory infiltration, and fibrosis of renal cortex and medulla.^14^^,^ 15 A combination of these factors when left untreated leads to kidney dysfunction overtime.

Posterior urethral valves (PUV) occurs in 1 in 5000 to 8000 live births and 1 in 1250 fetal ultrasonography screenings.^16^^,^ 17 PUV leads to bladder dysfunction with long-term morbidity and end-stage renal disease (ESRD) in 70% cases even after surgical treatment.^16^ It also leads to dilatation of ureters and obstruction or reflux at the vesico-ureteric junction (VUJ). Renal damage varies from reversible, less damaging obstructive uropathy to irreversible, severe renal dysplasia.^16^^,^ 17 VUR can be primary where the cause is unknown or secondary due to bladder obstruction or dysfunction. The higher the grade of VUR, more severe is the damage, leading to parenchymal injury, scarring, and dysfunction.^18^

The pSFK develops adaptive response as early as 22 weeks of intra-uterine life that progresses throughout childhood while sSFK develops compensatory changes after substantial loss of the contralateral kidney in a rapid manner.^19^ As the mature glomeruli have low mitotic activity, the compensatory changes lead to extensive hypertrophy of glomeruli without an increase in number. Thus, sSFK is vulnerable to additional stress compared to pSFK.^19^ Danton et al.^20^ found a 45% increase in nephron number in the remnant kidney when the contralateral kidney was removed in ovine fetuses. As the human nephrogenesis is similar to ovine species, that study suggests that pSFK produce effective compensatory mechanisms whereas sSFK does not. Our study shows that sSFK significantly increased the prevalence of renal damage as suggested by increased albuminuria compared to pSFK. This is supported by Pauline Abou et al.^19^

Ipsilateral CAKUT is seen in 25-45% of cases.^1^^,^ 21 We found ipsilateral CAKUT in 58% of cases. VUR is the most common ipsilateral CAKUT associated with SFK (37%), followed by VUJ obstruction (11 to 18%) and PUJ obstruction (6 to 7%).^18^ Our study supports this finding and demonstrates that ipsilateral CAKUT tends to worsen the outcome as the children with SFK and ipsilateral CAKUT had significantly higher rates of hypertension, reduced eGFR, CKD, and albuminuria, which are well supported by Westland et al^1^.

Thirty-eight percent of our study group had associated non-urological anomalies, which is consistent with studies by Kamal 21 (53%) and Dursun et al^22^ (44%). Anorectal malformation was the most common anomaly as found in other studies such as by Dursun et al.^22^

The prevalence of hypertension in our study was 24%. Hypertension was noted in 13% of cases by Westland et al.^1^ and 26% by Dursun et al.^22^ It was found that 91% of our study group had at least one of the markers of renal injury such as hypertension, microalbuminuria or reduced eGFR. We also found that the major part of the study group had compromised growth. CKD was seen in 60% of the study population, which is more than found in other studies such as by Sanna-Cherchi et al.^2^ (29.5%) and Kamal^21^ (20%).

The American Academy of Pediatrics has published recommendations for children with SFK that includes avoiding high contact/collision sports activities (boxing, basketball, diving, field hockey, football (tackle), ice hockey, martial arts, rugby etc.) as frequency of renal injuries are second only to frequency of head injuries and are more common in malformed kidneys.^23^

## CONCLUSION

The majority of children with SFK are prone to renal damage in early life, characterized by poor somatic growth, hypertension, albuminuria, and renal failure. Unilateral renal agenesis/aplasia is the most common cause of primary SFK and pelvi-ureteric junction obstruction is the most common cause of secondary SFK. Secondary SFK is more prone to damage than the primary SFK. SFK with ipsilateral CAKUT has a poorer outcome than SFK without ipsilateral CAKUT. The children with SFK require close follow up to monitor the development of complications, which need to be anticipated and treated aggressively to protect the single kidney.
